# Real-Time Hybrid Multi-Sensor Fusion Framework for Perception in Autonomous Vehicles

**DOI:** 10.3390/s19204357

**Published:** 2019-10-09

**Authors:** Babak Shahian Jahromi, Theja Tulabandhula, Sabri Cetin

**Affiliations:** 1 Mechanical and Industrial Engineering, University of Illinois, Chicago, IL 60607, USA; 2 Information and Decision Sciences, University of Illinois, Chicago, IL 60607, USA; theja@uic.edu

**Keywords:** environment perception, sensor fusion, LiDAR, road segmentation, fully convolutional network, object detection and tracking, extended Kalman filter, autonomous vehicle

## Abstract

There are many sensor fusion frameworks proposed in the literature using different sensors and fusion methods combinations and configurations. More focus has been on improving the accuracy performance; however, the implementation feasibility of these frameworks in an autonomous vehicle is less explored. Some fusion architectures can perform very well in lab conditions using powerful computational resources; however, in real-world applications, they cannot be implemented in an embedded edge computer due to their high cost and computational need. We propose a new hybrid multi-sensor fusion pipeline configuration that performs environment perception for autonomous vehicles such as road segmentation, obstacle detection, and tracking. This fusion framework uses a proposed encoder-decoder based Fully Convolutional Neural Network (FCNx) and a traditional Extended Kalman Filter (EKF) nonlinear state estimator method. It also uses a configuration of camera, LiDAR, and radar sensors that are best suited for each fusion method. The goal of this hybrid framework is to provide a cost-effective, lightweight, modular, and robust (in case of a sensor failure) fusion system solution. It uses FCNx algorithm that improve road detection accuracy compared to benchmark models while maintaining real-time efficiency that can be used in an autonomous vehicle embedded computer. Tested on over 3K road scenes, our fusion algorithm shows better performance in various environment scenarios compared to baseline benchmark networks. Moreover, the algorithm is implemented in a vehicle and tested using actual sensor data collected from a vehicle, performing real-time environment perception.

## 1. Introduction

**Figure 1:**
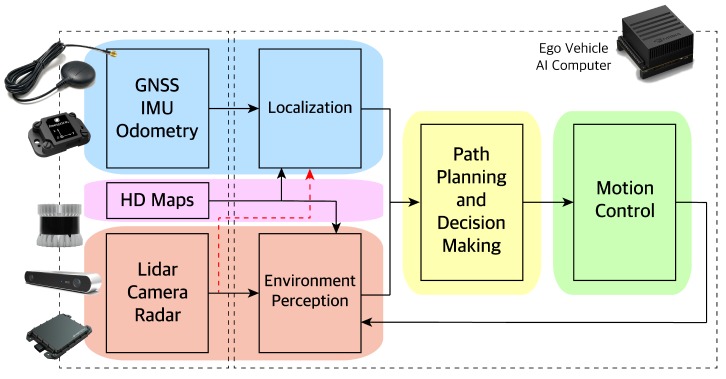
Autonomous vehicle architecture. Our paper focuses on the perception (red area).

Sensors in autonomous vehicles (AV) are used in two main categories: localization to measure where the vehicle is on the road and environment perception to detect what is around the vehicle. Global Navigation Satellite System (GNSS), Inertial Measurement Unit (IMU), and vehicle odometry sensors are used to localize the AV. Localization is needed, so the AV knows its position with respect to the environment. LiDAR, camera, and, radar sensors are used for environment perception. Understanding the surrounding is needed for the AV to navigate the environment safely. The output of localization and environment perception is sent to the AV path planner to decide on what actions to take. Those actions are sent to the AV motion control, which in turn will send the optimal control inputs to the actuators (accelerator, brakes, and steering). The AV architecture is shown in Figure 1.

In this research paper, we propose a hybrid multi-sensor fusion framework configuration for autonomous driving. In addition to accuracy improvement, this modular framework takes into account and combines the strengths of nonlinear state estimators i.e., Extended Kalman Filter technique with the strength of deep learning algorithms i.e., Fully Convolutional Network, based on the sensor type and configuration. This hybrid sensor fusion technique is used for understanding the environment around the autonomous vehicle to provide rich information on the safe driveable road region as well as detecting and tracking the objects on the road. This method was tested via an embedded in-vehicle computer, and the results were compared to the baseline networks and ground truth information.

There is numerous research on the environment perception for autonomous vehicles, including the sensors used in an AV, sensor data processing, and various fusion algorithms. The sensors can be categorized into three main categories: (1) camera; (2) LiDAR; and (3) radar. Also, the fusion algorithms can be categorized into two main categories: (1) sensor fusion using state estimators i.e., Kalman filters (KF); and (2) machine learning-based methods i.e., deep neural networks (DNN). Literature focus has been often on improving the algorithm accuracy performance; the implementation feasibility of these algorithms in an autonomous vehicle, however, has been less explored. The need for an efficient, lightweight, modular, and robust pipeline is essential. Therefore, a sensor fusion method configuration that balances a trade-off between fusion model complexity and real-world real-time applicability while improving the environment perception accuracy is fundamental.

The paper is structured as follows, we review the sensor fusion literature in Section 2. We review the related perception sensors technology (i.e., camera, LiDAR and radar) used in our implementation in Section 3. Next, we present our sensor fusion pipeline overview in Section 4, along with presenting how we processed the sensor data and implemented the fusion methods (i.e., details of our proposed FCNx and review of traditional EKF). In Section 5, the experiment procedure, tools and dataset is discussed. The experimental results and evaluation is presented in Section 6. We conclude our paper in Section 7.

## 2. Fusion Systems Literature Review

Reviewing related work on fusion systems [1,2,3,4,5], we can see that a lot focus has been given to camera and LiDAR sensors and performing different computer vision tasks using deep learning methods. Some literature focused on single sensor (camera or LiDAR) and single method (deep learning or traditional computer vision) techniques [6]. Some literature focused on LiDAR + camera multi-sensor approach [1,2,7] and a single fusion method. Other literature focused on camera + radar + LiDAR multi-sensor fusion using state estimators i.e., Kalman filters or particle filters [8,9,10]. Also, some works focused on the fusion method techniques without any real-world implementation or any specific set of sensors. In [11], the authors reviewed autonomous vehicle systems technology. They present the history of autonomous driver assistance systems and the sensor technology used in autonomous vehicles. They also explored standard sensor fusion methods as well as methodologies that connect the sensor information with actuation decision. In [12], a review of environment perception algorithms and modeling methods in intelligent vehicles is presented focusing on lane and road detection, traffic sign recognition, vehicle tracking, behavior analysis, and scene understanding. An overview of sensors and sensor fusion methods in autonomous vehicles is given [13]. In [6], an RGBD architecture is used for object recognition using two separate Convolutional Neural Networks (CNN). In [1,2], LiDAR and camera information is fused using a boosted decision tree classifier for road detection. Vision-based road detection is explored using geometrical transforms in [3]. Reference [4] proposed a unified approach to perform joint classification, detection, and semantic segmentation with a focus on efficiency. Deep learning-based road detection is developed using only LiDAR data and a fully convolutional network (FCN) in [14]. In [15], a high-level sensor data fusion architecture is designed and tested for ADAS applications such as emergency stop assistance. Reference [16] fused camera images and LiDAR point clouds to detect road using several FCNs with three different fusion strategies: early fusion, cross fusion, and late fusion. In [17], millimeter-wave radar and mono camera sensors are fused for vehicle detection and tracking. They present a fusion approach to achieve the optimal balance between vehicle detection accuracy and computational efficiency. Reference [18] presented a decoder-encoder FCN called SegNet for pixel-wise segmentation of road and indoor scenes images. In [7], a deep learning framework called ChiNet is proposed for sensor fusion using a stereo camera to fuse depth and appearance features on separate branches. ChiNet has two input branches for depth and appearance and two output branches for segmentation of road and objects. In [8], a fusion system is proposed based on a 2D laser scanner and camera sensors with Unscented Kalman filter (UKF) and joint probabilistic data association to detect vehicles. Reference [10] proposes a choice of fusion techniques using a Kalman filter, given a sensor configuration and its model parameters. Reference [9] investigated the problem of estimating wheelchair states in an indoor environment using UKF to fuse sensor data. A deep learning-based fusion framework for an end-to-end urban driving is proposed using LiDAR and camera [5]. A two-stage fusion method is used for hypothesis generation and verification using a stereo camera and LiDAR to improve vehicle detection in [19]. Real-time vehicle detection and tracking system are proposed for complex urban scenarios that fuse a camera, a 2D LiDAR and road context from prior road map knowledge using a CNN and a KF [20]. Reference [21] proposed a system in which a camera is used for pedestrian, bicyclist, and vehicle detection, which is then used to improve tracking and movement classification using radar and LiDAR. A multi-spectral pedestrian detection system is proposed using deep learning from a thermal camera and an RGB camera in [22]. In terms of architectures, Reference [23] proposed a convolutional neural network to perform semantic segmentation called fully convolutional network (FCN). Reference [24] improved this architecture by proposing the deconvolutional network (DeconvNet) by using decoding layers with deconvolutional filters that upsample the down-sampled feature maps. In SegNet [25], DeconvNet was improved by using connections between encoder and decoder layers. In U-Net, another encoder-decoder-based architecture for semantic segmentation all the feature maps are transferred from the encoding layers to the decoding layers via residual connections. Mask-RCNN [26] was also proposed in which it combines Faster-RCNN [27] object detection network with an FCN in one architecture. Our goal is to provide a cost-effective, lightweight fusion system solution that can be implemented in an relatively cheap and efficient embedded edge computer.

## 3. Autonomous Vehicle Perception Sensors

There are multiple sensors used for perception in autonomous cars that will cover the environment around the autonomous vehicle (AV). These sensors are: 1. Camera, 2. LiDAR (Light Detection and Ranging), 3. Radar (Radio Detection and Ranging), and 4. Sonar (Sound Navigation Ranging) to a lesser extent.

**Figure 2:**
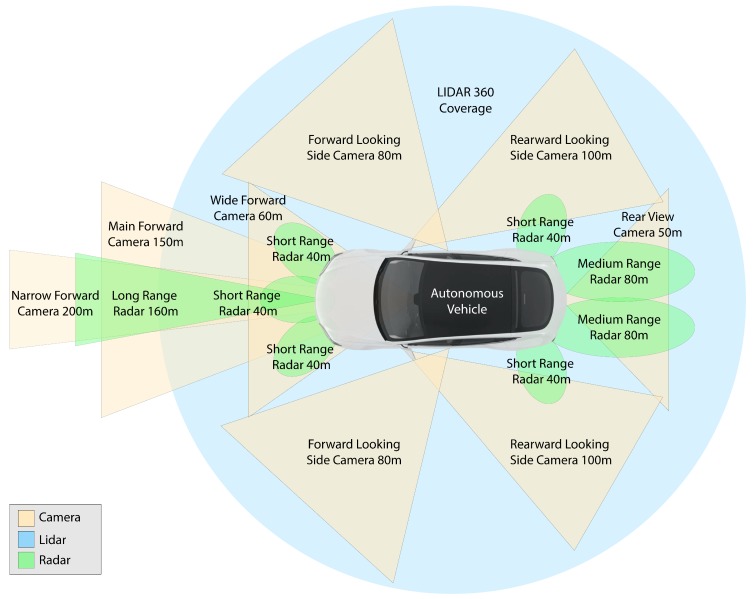
Surround sensors used for environment perception in autonomous vehicles. Their placement on the vehicle, coverage, and maximum range is shown. Green areas show radars coverage (long-range, medium-range and short-range), orange areas show camera coverage around the car, blue indicates LiDAR 360-degree coverage.

In this paper, we stick with three primary sensors: camera, LiDAR, and radar. The main sensor’s placement on a typical autonomous vehicle, their coverage and max range are shown in Figure 2. A camera is affected by environmental variations such as occlusions, illumination and weather variation; LiDAR is affected by bad weather such as snow, rain; and radar has low detection accuracy and resolution. Using these complementary sensors with the appropriate sensor fusion method suited for them addresses the ineffectiveness of each sensor. It also increases the fault tolerance and accuracy of the entire perception framework.

### 3.1. Camera

**Figure 3:**
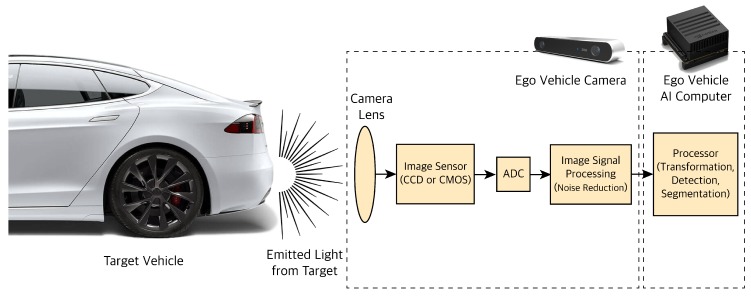
Camera sensor processing pipeline.

Cameras are the primary sensors used for high-resolution tasks like object classification, semantic image segmentation, scene perception, and tasks that require color perception like traffic lights or signs recognition. Cameras work on the basic principle of detecting the light emitted from the objects on a photosensitive surface through a lens, as shown in Figure 3. The photosensitive surface, also called the image plane, is where the light is stored as an image. After light photons pass through a camera lens, they hit a light-sensitive surface which creates several electrons. The photosensitive surfaces measure the amount of light they receive and convert that into electron movements. Those moving electrons form a charge which is then converted to a voltage by a capacitor. The voltage is amplified and passed through an Analog-Digital Converter (ADC). The ADC converts the amplified voltage to a digital number i.e., a digital gray value (pixels) that is related to the intensity of light. The Image Signal Processor (ISP) enhances the quality of the noisy image captured. Image noises source from imperfect lenses, filters and image sensors. Image sensors are color blind by default and cannot differentiate between different wavelengths. Therefore, we use microlenses or filters in front of each pixel to only allow a certain wavelength. One filter pattern to map wavelength to color is the RGB pattern. The most common RGB pattern is the Bayer pattern or Bayer filtering. The position of the object can be defined as a vector with three elements [x, y, z] in space which is going to get projected through a lens to a point on the image plane [x’, y’]. The projected points are converted from the metric unit to pixels so the image can be used for further processing and extracting information such as detection, classification, tracking of objects that are in the path of our autonomous vehicle (ego vehicle).

Before using the camera sensors for our implementation and testing applications, we calibrate each camera; this is due to the use of lenses in the camera sensors. Camera lenses distort the images they create. We rectified these distortions by using pictures of known objects i.e., checkerboards. The dimensions and geometry of the checkerboards are known; therefore, we can compute the calibration parameters by comparing the camera output images with the original images to account for those distortions. There are two major camera chips in the market today, namely: Charge-Coupled Device (CCD) and Complementary Metal Oxide Semiconductor (CMOS) [28]. There is also new camera technology developed called Live-MOS which combines the strengths of CCD and CMOS. In CCD chip the photosites (RGB blocks) are passive, and the electrons at those sites are processed one column at a time. The Amplification and A/D conversion also happen outside of the sensor. In CMOS, as opposed to CCD the photosites are active, and each includes its amplifier and ADC. We used a CMOS camera for our application due to the lower cost, lower power consumption, and faster data readout. The advantage CCD has over CMOS is its high light sensitivity, which results in better image quality at the cost of higher power consumption that can lead to heat issues and a higher price.

### 3.2. RADAR

**Figure 4:**
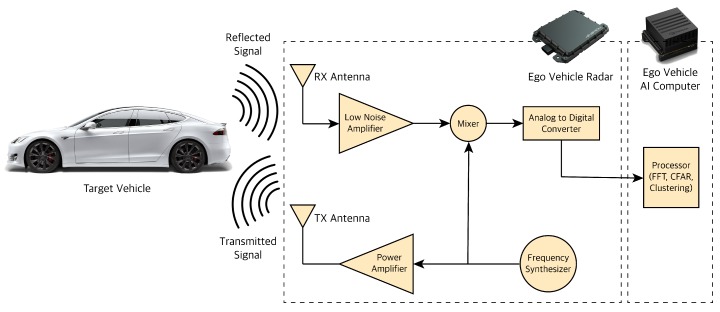
Radar sensor processing pipeline.

Radar stands for Radio Detection and Ranging. Radar works on the principle of transmitting and detecting electromagnetic (EM) radio waves. Radars can measure information about the obstacles they detect like relative position based on the wave travel time and relative velocity based on the Doppler property of EM waves. The frequency of radars EM waves are not affected by different lighting or weather conditions; thus, they can detect at day or night, in foggy, cloudy, or snowy conditions. Automotive radars are usually mounted either behind a bumper or in the vehicle grille; the vehicles are designed to ensure the radar performance (the radar antenna ability to focus EM energy) is not affected no matter where they are mounted. For automotive applications, Frequency-Modulated Continuous Wave (FMCW) type radar is typically used [29]. FMCW radar transmits continuous waves or chirps in which the frequency increases (or decreases) over time usually in a sawtooth waveform. These radars also typically work at frequencies of 24 GigaHertz (GHz), 77 GHz and 79 GHz. The GHz frequency corresponds to millimeter wavelengths; hence, they are also called Millimeter Wave (MMW) radars. There are three major classes of automotive radar systems depending the application: SRR (Short-Range Radar) mostly for parking assist and collision proximity warning, MRR (Medium-Range Radar) mostly for blind-spot detection, side/rear collision avoidance and LRR (Long-Range Radar) for adaptive cruise control and early collision detection mounted upfront. FMCW radar hardware consists of multiple components as shown in Figure 4.

The Frequency Synthesizer generates the FMCW wave or chirp at the desired frequency. The wave is then amplified using a power amplifier depending on the radar class and the desired distance coverage for the radar. The further the desired range, the higher the power amplification needed for the signal. The transmit (TX) antenna converts the electrical energy of the signals to electromagnetic signals and transmits them through the air. The receive (RX) antenna receives the reflected signal. Field of View (FoV) of radar is determined by the antenna type, pattern, and gain value, discussing which is out of the scope of this paper. The antenna also increases the power of the signal by focusing the energy in a specific direction. The received signal is amplified through a Low Noise Amplifier (LNA) since some of the EM energy is attenuated by moving through air and the targets it hits during the signal travel. The amplified received signal is sent to a mixer. Mixer multiplies the reflected signal by the original signal generated by the synthesizer. Through this operation, mixer changes the frequency of the EM wave to the difference of the original signal and reflected signal frequency. The mixer will then output the delta frequency, also known as frequency-shift or intermediate frequency (IF). The output of the mixer is converted from analog to digital using an analog digital converter (ADC) so we can further process the signal for target detection and tracking.

### 3.3. LiDAR

**Figure 5:**
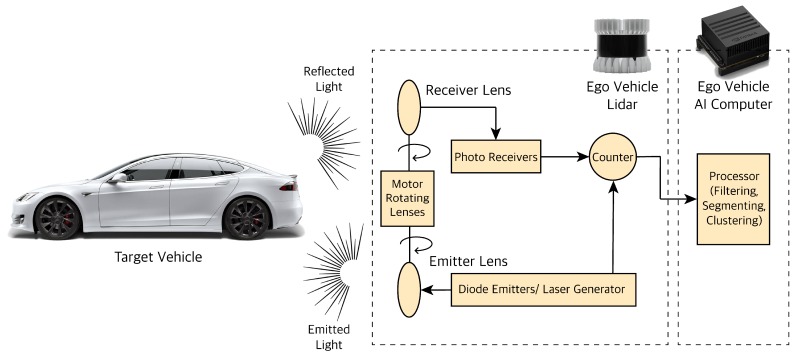
LiDAR sensor processing pipeline.

LiDAR stands for Light Detection and Ranging. LiDAR works on the principle of emitting a laser light or Infrared (IR) beam and receiving the reflection of the light beams to measure the surrounding environment. The LiDAR used for autonomous vehicles are mostly in the 900 nanometer (nm) wavelength, but some have a longer wavelength which works better in fog or rain. LiDAR outputs point cloud data (PCD) which contain the position (x, y, z coordinates) and intensity information of the objects. Intensity value shows the object’s reflectivity (the amount of light reflected by the object). The position of the objects is detected using the speed of light and time of flight (ToF). There are various classes of LiDAR. The two major ones are Mechanical LiDAR and Solid State LiDAR (SSL). In Mechanical/Spinning LiDAR, the laser is emitted and received at the environment using rotating lenses driven by an electric motor to capture a desired FoV around the ego vehicle. Solid State LiDARs (SSL) do not have any spinning lenses to direct the laser light; instead, they steer the laser lights electronically. These LiDARs are more robust, reliable, and less expensive than the mechanical counterparts, but their downside is their smaller and limited FoV compared the mechanical ones. It is worth noting that the laser beams generated in LiDARs are class 1 laser or the lowest intensity out of four categories of laser classes; therefore, their operation is considered harmless for the human eyes. Typical mechanical LiDAR hardware consists of various components as shown in Figure 5.

The laser light is generated and emitted using diode emitters; it is passed through rotating lenses to focus its energy at various angles. The light is then reflected off the objects and received through another set of rotating lenses. Reflected light information is received through photoreceivers. The timer/counter then calculates the ToF between the emitted and received laser beams to measure the object position state information. The resulting point cloud information is then sent to an on-board vehicle processor for further processing. LiDAR adds angular resolution and higher accuracy to the camera and radar sensors.

Similar to cameras, LiDAR captures 2D (X-Y direction) spatial information; however, unlike cameras it captures ranges to create a 3D spatial information of environment around the ego vehicle. By measuring range, LiDAR adds angular resolution (horizontal azimuth and vertical) for better measurement accuracy compared to the camera. Also, having a higher frequency and shorter wavelengths enables LiDAR to be more accurate than radar sensor measurements. Typical LiDAR has a range of about 40 to 100 m, resolution accuracy between 1.5–10 cm, vertical angular resolution between 0.35 to 2 degrees, horizontal angular resolution of 0.2 degrees and an operating frequency of 10–20 Hz.

## 4. Hybrid Sensor Fusion Algorithm Overview

**Figure 6:**
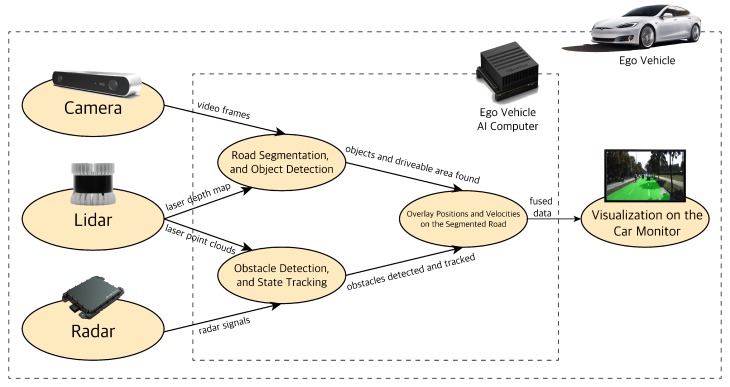
Multi-sensor fusion algorithm pipeline.

The three main perception sensors used in autonomous vehicles have their strengths and weaknesses. Therefore, the information from them needs to be combined (fused) to make the best sense of the environment. Here, we propose a hybrid sensor fusion algorithm to this end. The hybrid sensor fusion algorithm consists of two parts that run in parallel as shown in Figure 6. In each part, a set configuration of sensors and a fusion method is used that is best suited for the fusion task at hand.

The first part deals with high-resolution tasks of object classification, localization, and semantic road segmentation by using the camera (vision) and LiDAR sensors.

The camera’s raw video frames and the depth channel from the LiDAR are combined before being sent to a Deep Neural Network (DNN) in charge of the object classification and road segmentation tasks. Deep networks such as Convolutional Neural Networks (CNN) and Fully Convolutional Networks (FCN) have shown better performance and accuracy for computer vision (CV) tasks compared to traditional methods i.e., feature extractors (SIFT, HOG) with a classic Machine Learning (ML) algorithm (SVM) [30,31].

The combination of camera and LiDAR with an FCN architecture gives us the best sensor-method combination for performing classification and segmentation. In this paper, we prioritize real-time performance and accuracy. Therefore, instead of shooting for segmenting the road scene into multiple small fine segments as has often been done in the literature [32], we only perform segmentation for two classes: the free space (driveable area) of the road and not driveable area of the road for our autonomous vehicle. We can also use an efficient and fast network like YOLO V3 [33] to detect obstacles within the segmented road. For automated driving, knowledge of driveable space and obstacle classes on the road is essential for path planning and decision making.

The second part deals with the task of detecting objects and tracking their states by using LiDAR and radar sensors. The LiDAR point cloud data (PCD) and radar signals are processed and fused at the object level. The LiDAR and radar data processing will result in clusters of obstacles on the road within the region of interest (ROI) with their states. The fusion of processed sensor data at the object level is referred to as late fusion. The resulting late fused LiDAR and radar data is sent to a state estimation method to best combine the noisy measured states of each sensor. The Kalman Filter (KF) is the method of choice for state estimation; however, they work on the assumption of linear motion models. Since the motion of obstacles like cars is non-linear; we use a modified version of the KF, namely the Extended Kalman Filter (EKF). Knowing and tracking the states of the obstacles on the road helps to predict and account for their behavior in the AV path planning and decision making stacks. Finally, we overlay each fusion output and visualize them on the car monitor.

### 4.1. Object Classification and Road Segmentation Using Deep Learning

For road semantic segmentation, we use camera images as well as depth information from the LiDAR. We combine these raw data at the depth channel, which results in an RGBD image with a depth channel of size four. We then send this information to our proposed Fully Convolutional Network (FCNx). FCNx is an encoder-decoder-based network with modified filter numbers and sizes in the downsampling and upsampling portions and improved skip connections to the decoder section. The model is trained and tested on actual sensor data on our embedded NVIDIA GPU-powered computers. Finally, the model performance is evaluated by comparing its predictions with baseline benchmark networks and the ground truth labels.

#### 4.1.1. Camera-LiDAR Raw Data Fusion

**Figure 7:**
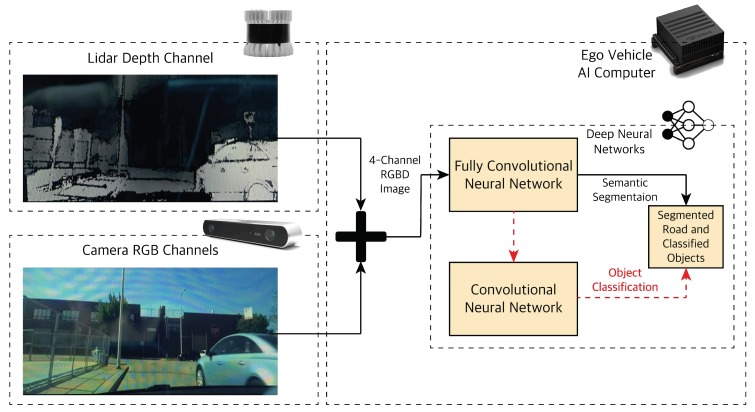
Early fusion of LiDAR depth image and camera image as an input to a DNN pipeline.

Before diving into the FCN architecture; we need to prepare the data that feeds into it. The camera and LiDAR sensors provide the input data for the FCN Figure 7. The camera provides 2-dimensional (2D) color images of three RGB channels (Height × Width × 3). The LiDAR provides a high-resolution depth map in addition to the point cloud data. We combine the unprocessed raw data of LiDAR and camera (early fusion). The resulting RGBD image will have four channels (H × W × 4). The RGBD image contains the 2D appearance features of the camera image with 3D depth features of LiDAR to give us a rich illumination-invariant spatial image. This image is fed to the FCN to extract road features for semantic segmentation and a CNN for object detection and localization depending on the application.

#### 4.1.2. Proposed Fully Convolutional Network (FCNx) Architecture

The architecture consists of two main parts: an encoder and a decoder. First, we have the encoder; it is based on the VGG16 architecture [34], with its fully connected layers replaced with convolutional layers. The purpose of the encoder is to extract features and spatial information from our four-channel RGBD input image. It uses a series of convolutional layers to extract features and max-pooling layers to reduce the size of the feature maps. Second, we have a decoder, which is based on the FCN8 architecture [23]. Its purpose is to rebuild the prediction of pixel classes back to the original image size while maintaining low-level and high-level information integrity. It uses transposed convolutional layers to upsample the output of the last convolutional layer of the encoder [24]. Also, it uses skip convolutional layers to combine the finer low-level features from encoder layers with the coarser high-level features of transposed convolutional (upsampled) layers.

**Figure 8:**
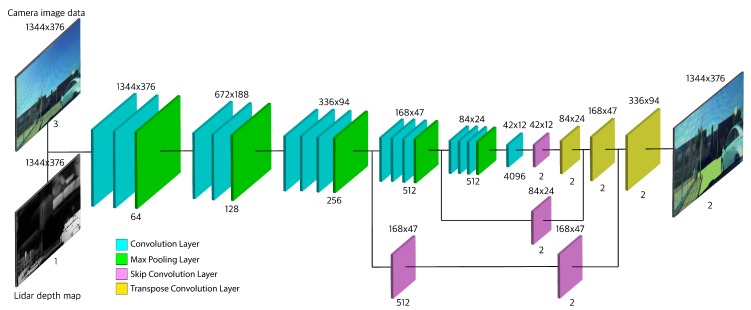
Overview of the object detection and road segmentation architecture via a Fully Convolutional Neural Network. The tiles and colors indicate different layers of the FCNx and their type respectively. Encoder consists of: convolution (teal) and max pooling (green) layers. Decoder consists of: skip convolution (purple) and transposed convolution (yellow). The size and number of feature maps are shown at the top and bottom of each layer.

In our proposed network, which builds on the above shown in Figure 8, we combine the encoder output from layer four with the upsampled layer seven. This combination is an element-wise addition. This combined feature map is then added to the output of the third layer skip connection. In the skip connection of the layer three output, we use a second convolutional layer to further extract features from layer three output. The addition of this convolutional layer adds some features that would be extracted in layer four. Including some basic layer four level feature maps will help the layer three skip connection to represent a combined feature map of layer three and layer four. This combined feature map is then added to the upsampled layer nine, which itself represents a combined feature map of layer seven and layer four. This addition is shown to give a better accuracy and lower Cross-Entropy loss compared to the base VGG16-FCN8 architecture. The better performance can be explained by the fact that having some similar layer four feature maps can help better align the extracted features when performing the last addition. We will refer to our proposed architecture as FCNx.

The purpose of the FCNx applied to camera and LiDAR fused raw data is to segment the road image into free navigation space area and non-driveable area. The output can be further processed and sent through a plug and play detector network (YOLO [33,35,36], SSD [37]) for object detection and localization depending on the situation.

### 4.2. Testing Obstacle Detection and Tracking using Kalman Filtering

In this section, we use the radar and LiDAR sensors to detect obstacles and measure their states by processing each sensor data i.e., the radar beat signal and LiDAR point cloud individually. We then perform a late data fusion or an object-level fusion to add the processed data from the radar and LiDAR. Then, using a non-linear Kalman Filter method, we take those noisy sensor measurements to estimate and track obstacle states with a higher accuracy than each sensor.

#### 4.2.1. Radar Beat Signal Data Processing

**Figure 9:**
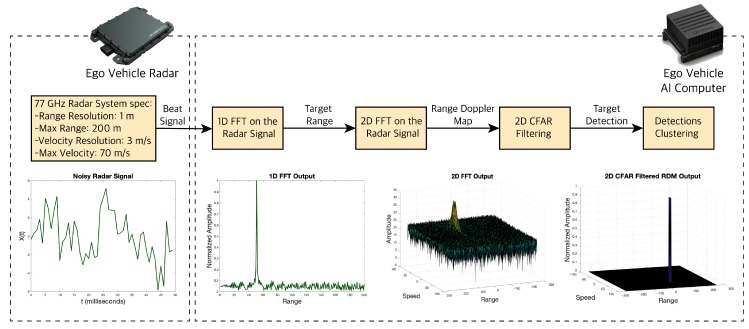
Radar data processing pipeline. Plots from left to right: Noisy radar beat signal, output of the 1D-FFT showing a detection with its range, output of the 2D-FFT showing a detection with its range and Doppler velocity, and output of the 2-Dimensional Constant False Alarm Rate (2D-CFAR) filtering showing the detection with noises filtered out.

The radar sensor can detect obstacles and their states in a four step process as shown in Figure 9. The first step is to process the noisy digitized mixed or beat signal we receive from the radar. The beat signal is sent through an internal radar ADC to get converted to a digital signal. The digital signal is in the time domain, which comprises of multiple frequency components. In the second step, we use a one-dimensional (1D) Fast Fourier Transform (FFT), also known as 1st stage or Range FFT, to transform the signal from the time domain to frequency domain, and separate all its frequency components. The output of the FFT is a frequency response represented by signal power or amplitude in dBm unit versus beat frequency in MHz unit. Each peak in the frequency response represents a detected target.

The x-axis can be converted from beat frequency of the targets to range of the targets by:

(1) $$R=\frac{c\cdot T_{sweep}\cdot f_{b}}{2\cdot B_{sweep}}$$

where *R* is the range of the target, *c* is the speed of light and $f_{b}$ is the beat frequency of the target. The $T_{sweep}$ and $B_{sweep}$ are the chirp/sweep time and bandwidth respectively, which can be calculated by:

(2) $$T_{sweep}=5.5\cdot \frac{2\cdot R_{max}}{c}$$

(3) $$B_{sweep}=\frac{c}{2\cdot d_{res}}$$

where $R_{max}$ is the radar maximum range and the $d_{res}$ is the range resolution of the radar. The constant 5.5 comes from assumption that the sweep time is often between 5–6 times the delay corresponding to the maximum radar range.

As discussed, the range FFT output gives us the beat frequency, amplitude, phase, and range (from the equations above) of the targets. To measure the velocity (or Doppler velocity) of the targets, we need to find the Doppler frequency shift, which is the rate of change of phase across radar chirps. The target phase changes from one chirp to another. Therefore, after acquiring the range FFT on the radar chirps, in the third step, we run another FFT (Doppler FFT) to measure the rate of change of phase i.e., the Doppler frequency shift. The output of the Doppler FFT is a 3D map represented by signal power, range, and Doppler velocity. This 3D map is also referred to as Range Doppler Map (RDM). RDM gives us an overview of the targets range and velocity.

**Figure 10:**
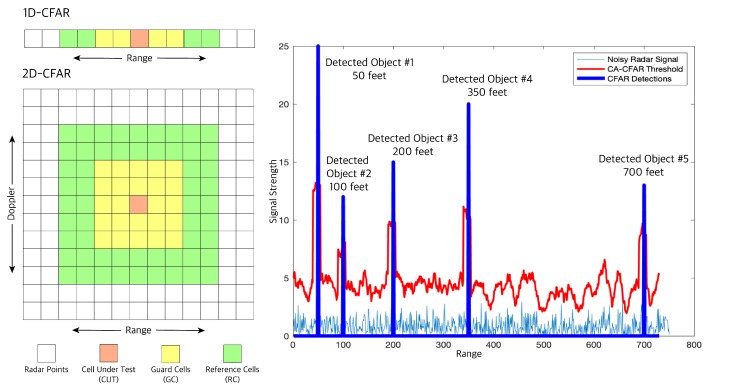
1D and 2D-CFAR sample windows (left) with 1D-CFAR multi object detection (right). The plot shows signal strength vs. range. Five objects are detected (the blue peaks) at ranges 50, 100, 200, 350 and 700 feet.

RDM can be quite noisy since reflected radar signals received can be from unwanted sources like the ground, and buildings, which can create false alarms in our object detection task. In the fourth and final step, these noises or clutters are filtered out to avoid such false positives. One filtering method most used in automotive applications is Cell Averaging CFAR (CA-CFAR) [38] which is a dynamic thresholding method i.e., it varies thresholds based on the local noise level of the signal, see Figure 10. CA-CFAR slides a window across the 2D FFT output cells we generate. The sliding window in a 2D CFAR includes: the Cell Under Test (CUT) or current cell tested for target presence, the guard cells (GC) or cells around the CUT that prevent the leakage of the target signal on to the surrounding cells hence negatively affecting the noise estimate, and finally the reference or training cells (RC) or cells covering the surrounding of the CUT. The local noise level is estimated by averaging the noise under the training cells. If the signal in CUT is smaller than the threshold, it is removed. Anything above the threshold is considered a detection.

Finally, we have radar CFAR detection with range and Doppler velocity information but object detection and tracking in real time, is a computationally expensive process. Therefore, we cluster the radar detection that belongs to the same obstacle together to increase the performance of our pipeline. We use a Euclidean distance-based clustering algorithm. In this method, all the radar detection points that are within the size of the target are considered one cluster. The cluster is assigned with a range and velocity at the center equal to the mean of the ranges and velocities of all the cluster detection.

#### 4.2.2. LiDAR Point Cloud Data Processing

**Figure 11:**
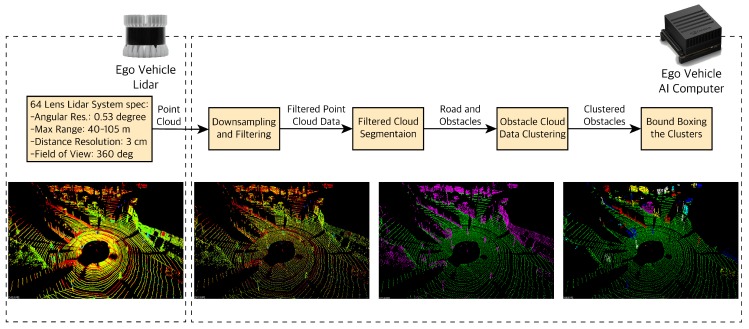
LiDAR data processing pipeline. Images from left to right: Dense LiDAR point cloud data, downsampled and filtered LiDAR data, segmented point cloud into road and obstacles, clustered obstacles in the segmented point cloud. The center dark spot is the ego vehicle.

LiDAR gives us rich information with point clouds (which include position coordinates x, y, z and intensity i) as well as a depth map. The first step to process the LiDAR point cloud data (PCD) as shown in Figure 11 involves downsampling and filtering the data. The raw LiDAR point cloud is high resolution and covers a long distance (for example a 64-lens laser acquires more than 1 million points per second). Dealing with such a large number of points is very computationally expensive and will affect the real-time performance of our pipeline. Therefore, we downsample and filter. We downsample using a voxel grid; we define a cubic voxel within the PCD and only assign one point cloud per voxel. After downsampling, we use a region of interest (ROI) box to filter any PCD outside of that box that is not of our interest (i.e., points from non-road objects like buildings). In the next step we segment the filtered PCD into obstacles and the road. Knowledge of the road and objects on the road are the two segments most important for the automated driving task. The PCD segmentation happens at the point level; hence, processing would require a lot of resources and slow down the pipeline. In order to improve the performance of our pipeline, similar to the radar data, we cluster the obstacle segments based on their proximity to neighboring points (Euclidean Clustering) and assign each cluster with a new position coordinates (x, y, z) which is the mean of all the point clouds within that cluster. Finally, we can define bounding boxes of the size of clusters and visualize the obstacles with the bounding boxes.

For segmenting the PCD into road and obstacles, we need to separate the road plane from the obstacle plane. In order find the road plane, we use the Random Sample Consensus (RANSAC) method [39]. Using RANSAC, we fit a plane to our 3D point cloud data. At each step, RANSAC picks a sample of our point clouds and fits a plane through it. We consider the road points as inliers and obstacle points as outliers. It measures the inliers (points belonging to the road plane) and returns the plane i.e., road plane with the highest number of inliers or the lowest number of outliers (obstacles).

#### 4.2.3. Extended Kalman Filtering

After the semantic road segmentation using our FCNx network, in this section, we show the implementation of single object tracking via our vehicle embedded computer using the traditional Extended Kalman Filter (EKF) algorithm. This is to showcase the real-world implementation of object tracking, predicting and maintaining their states (2-dimensional positions and velocities) over time. State of the object being tracked are: $P_{x}$, $P_{y}$ (the object’s positions in x and y-direction), and $V_{x}$, $V_{y}$ (the object’s velocities in x and y-direction). For state tracking, we use a state estimation method called Kalman filtering (KF).

A standard KF involves three steps: initialization, prediction, and update. For the prediction step, we use the object’s current state ${\hat{x}}_{k}$ (2D-position and 2D-velocity) and assume that the object has a constant velocity (CV) motion model, to estimate the state at the next time step ${\hat{x}}_{k+1}$ using state transition function F in Equation (4). Object covariance P represents the state estimate uncertainty in Equation (5). We also model the noise in KF, the state transition noise $\hat{\xi }$, motion noise covariance Q, measurement noise covariance R. After getting the measurement from our LiDAR or radar sensor, we map the state to the actual measured value of the sensors z using the measurement function H using Equation (6). we calculate the residual covariance from Equation (7).

(4) $${\hat{x}}_{k+1}=F\cdot {\hat{x}}_{k}+\hat{\xi }$$

(5) $$P_{k+1}=F\cdot P_{k}\cdot F^{T}+Q$$

(6) $${\hat{y}}_{k+1}={\hat{z}}_{k+1}-H\cdot {\hat{x}}_{k+1}$$

(7) $${\hat{s}}_{k+1}=H\cdot P_{k+1}\cdot H^{T}+R$$

Kalman gain *K*, which combines the uncertainty of our predicted state $P_{k+1}$ with the uncertainty of sensor measurements $S_{k+1}$. Kalman gain will give more weight on the less uncertain value; either the predicted state ${\hat{x}}_{k+1}$ or the sensor measurement ${\hat{z}}_{k+1}$. We calculate the updated state estimate as well as its covariance from Equations (9) and (10).

(8) $$K=P_{k+1}\cdot H^{T}\cdot {S_{k+1}}^{-1}$$

(9) $${\hat{x}}_{k+1}={\hat{x}}_{k}+K\cdot \hat{y}$$

(10) $$P_{k+1}=\left(I-K\cdot H\right)\cdot P_{k}$$

The standard Kalman filter can only be used when our models are linear (motion model or the measurement model.) with the assumption that our data has a Gaussian distribution. When data is Gaussian and motion-based, and measurement models are linear, the output data will also have Gaussian distribution. However, in a system, the motion model or measurement model or both can be nonlinear. When either or both these models do not follow a Gaussian distribution and are nonlinear, the input states will go through a nonlinear transformation; Hence, the standard KF may not converge, and its equations are not valid. In the case of nonlinear models, we have to use a nonlinear state estimator like an Extended Kalman filter (EKF). EKF fixes this problem by linearizing the nonlinear functions (state transition and measurement functions) around the mean of the current state estimate using Taylor series expansion and taking the Jacobian of our functions [40]. In EKF, the system equations are:

(11) $${\hat{x}}_{k+1}=f\left({\hat{x}}_{k},{\hat{u}}_{k}\right)+\hat{\xi }$$

(12) $${\hat{z}}_{k+1}=h\left({\hat{x}}_{k+1}\right)+\hat{\epsilon }$$

where $f({\hat{x}}_{k},{\hat{u}}_{k})$ and $h({\hat{x}}_{k+1})$ are the nonlinear state transition and measurement functions respectively. We linearize these functions by finding their Jacobians, so the linearized approximated system becomes:

(13) $${\hat{x}}_{k+1}=F\cdot {\hat{x}}_{k}+\hat{\xi }$$

(14) $${\hat{z}}_{k+1}=H\cdot {\hat{x}}_{k+1}+\hat{\epsilon }$$

where *F* and *H* are approximated as: $F=\frac{\partial f}{\partial x}$ and $H=\frac{\partial h}{\partial x}$.

For our tracking implementation, we use two sensor measurements: LiDAR and radar. From LiDAR point cloud data, we acquire the object’s timestamped position in *x* and *y* directions: $P_{x}$, $P_{y}$. From radar data, we acquire the objects timestamped position in *x* and *y* directions: $P_{x}$, $P_{y}$ as well as the objects timestamped velocity in *x* and *y* directions: $V_{x}$, $V_{y}$. LiDAR sensor measurements are in Cartesian coordinates therefore linear. Radar sensor measurements are typically in polar coordinates (unless a separate radar signal processing is done), i.e., timestamped ρ, the radial distance from the sensor to the detected objects, ϕ, the angle between the radar beam and the x-axis as well as $\dot{\rho }$, the radial velocity along the radar beam.

For this nonlinear case we need to map our predicted state vector from Cartesian to polar coordinates using a nonlinear measurement function $h({\hat{x}}_{k+1})$; in order to linearize the measurement function, we take its Jacobian using Equation (15):

(15) $$H=\left[\begin{array}{cccc} \frac{P_{x}}{\sqrt{{\left(P_{x}\right)}^{2}\;+\;{\left(P_{y}\right)}^{2}}} & \frac{P_{y}}{\sqrt{{\left(P_{x}\right)}^{2}\;+\;{\left(P_{y}\right)}^{2}}} & 0 & 0\\ -\frac{P_{y}}{{\left(P_{x}\right)}^{2}\;+\;{\left(P_{y}\right)}^{2}} & \frac{P_{x}}{{\left(P_{x}\right)}^{2}\;+\;{\left(P_{y}\right)}^{2}} & 0 & 0\\ \frac{P_{y}\cdot \left(V_{x}\cdot P_{y}\;-\;V_{y}\cdot P_{x}\right)}{{\left({\left(P_{x}\right)}^{2}\;+\;{\left(P_{y}\right)}^{2}\right)}^{1.5}} & \frac{P_{x}\cdot \left(V_{y}\cdot P_{x}\;-\;V_{x}\cdot P_{y}\right)}{{\left({\left(P_{x}\right)}^{2}\;+\;{\left(P_{y}\right)}^{2}\right)}^{1.5}} & \frac{P_{x}}{\sqrt{{\left(P_{x}\right)}^{2}\;+\;{\left(P_{y}\right)}^{2}}} & \frac{P_{y}}{\sqrt{{\left(P_{x}\right)}^{2}\;+\;{\left(P_{y}\right)}^{2}}} \end{array}\right]$$

where $P_{x}$ and $P_{y}$ are the object’s positions in x and y-direction; $V_{x}$ and $V_{y}$ are the object’s velocities in x and y-direction. We use these measurement values in the Extended Kalman filter algorithm.

## 5. Experiments Procedure Overview

For our FCNx architecture, we use a combined dataset for training. The dataset is a combination of UC Berkeley DeepDrive (BDD) dataset [41], University of Toronto KITTI dataset [42] and a self-generated dataset generated from our sensors installed in our test vehicle. We validated the proposed algorithm on a dataset with 3000 training and 3000 testing road scene samples. Our sensor data was acquired from a ZED camera and an Ouster LiDAR mounted on our test vehicle capturing data from streets of the city of Chicago. The BDD and KITTI dataset are annotated and labeled at object and pixel levels. For our dataset, we performed the annotating and labeling manually. We use these annotations as the ground truth labels. For our annotations, we mark the road with the color yellow.

**Figure 12:**
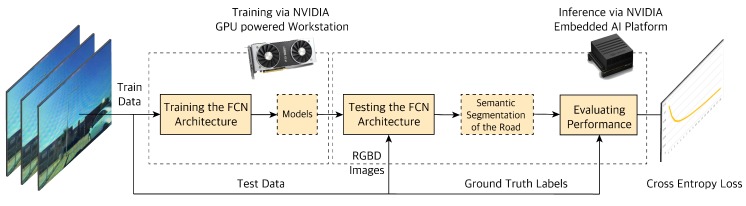
Overview of the experiment process. Training a Deep Neural Network model requires powerful computing resources; hence, for training we use a GPU powered workstation. Inference is less computationally expensive; thus, when the model is trained, we use an embedded GPU enabled device for testing and inference at the edge in-vehicle.

We trained the network by using the following tunable hyper-parameters. The parameter values are found based on trial and error with different values and combinations that give the best performance. Some of the hyper-parameters we picked are as follows: learning rate of $2\times 10^{-4}$, batch size of 5 and the keep probability of 0.5. We train the network using an NVIDIA GeForce RTX2080 Ti Ubuntu 18.04.3 LTS machine with 64 GB memory; then upload the model to an embedded NVIDIA Xavier edge computer in our vehicle for real-time inference. From the obtained results of our network we calculate the Cross-Entropy (C.E.) loss. This metric gives a measure of how well our segmentation network is performing. We define our performance goals as C.E. loss, using validation and strive to improve upon other baseline benchmarks networks namely FCN8 [23] and U-Net [43]. Another important factor for automated driving is performing inference in real time. Our network experiment procedure is shown in Figure 12.

There are different metrics to measure the performance of our free navigation space segmentation network like pixel accuracy, mean Intersect over Union (mIoU) and the Cross-Entropy (C.E.) loss. The most common loss function is a pixel-wise Cross-Entropy. In this loss, we compare each pixels predicted class to the ground truth image pixel labels. We repeat this process for all pixels in each image and take the average. In our segmentation we have two classes (i.e., binary), road and not-a-road. The Cross-Entropy loss is defined as:

(16) $$C.E.Loss=-(p\cdot \log\left(\hat{p}\right)+\left(1-p\right)\cdot \log\left(1-\hat{p}\right))$$

where the probability of pixel ground-truth values $y_{true}$ for the road defined as $P(Y_{true}=road)=p$ and for not-a-road defined as $P(Y_{true}=notroad)=1-p$. The predicted values $y_{pred}$ for road and not-a-road are defined as $P\left(Y_{pred}=road\right)=\hat{p}$ and $P\left(Y_{pred}=notroad\right)=1-\hat{p}$.

To show the implementation of single object tracking via the traditional EKF on our embedded edge computer, we use the LiDAR and radar sensor measurements ($P_{x}$ and $P_{y}$: the object’s positions in x and y-direction; $V_{x}$ and $V_{y}$: the object’s velocities in x and y-direction.) We use these measurement values in the Extended Kalman filter algorithm. For our testing purposes, the ground truth (the actual path of the object) is measured manually and used for calculating root mean square error (RMSE) from Equation (17) to measure single object tracking algorithm performance.

(17) $$R.M.S.E.=\sqrt{\frac{1}{n}\cdot \sum_{i=1}^{n}{{\left|\right|{\hat{x}}_{estimate}-{\hat{x}}_{groundtruth}\left|\right|}_{2}}^{2}}$$

where ${\hat{x}}_{estimate}$ is the EKF predicted states and ${\hat{x}}_{groundtruth}$ is the actual states of the test vehicle.

## 6. Experimental Results and Discussion

The results of the hybrid sensor fusion framework are presented in this section. We measure the performance using an evaluation metric appropriate for the fusion method; for our FCNx road segmentation we use the Cross-Entropy (CE) loss metric. We also show the results of implementing single object tracking using a traditional EKF fusion on our embedded edge computer. For tracking evaluation we use the root mean square error (RMSE) metric. We compare our proposed network architecture with two benchmarks FCN8 [23] and U-Net [43] networks architectures in Table 1.

**Figure 13:**
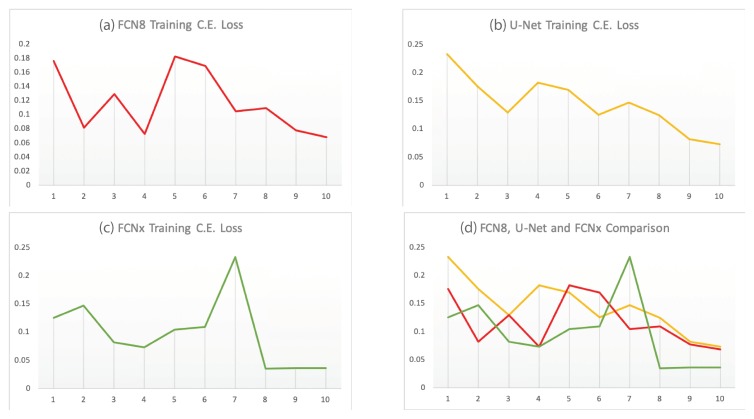
Comparison of FCN8 (**a**), U-Net (**b**), and FCNx (**c**) architectures training C.E. Loss over 10 training cycles (**d**).

Analytically, we compare our proposed network performance with two benchmarks FCN8 [23] and U-Net [43] networks performance by measuring the Cross-Entropy loss and inference time metrics shown in Table 2. Under the same conditions, i.e., the same training input data, hyperparameter choices and computing power; our model FCNx reduces the CE loss by 3.2% compared to FCN8 model while maintaining similar inference time. We also see an improvement of 3.4% over U-net model; although the U-net network showed faster inference time but the segmentation accuracy improvement of FCNx offers a better trade off than the inference time. Table 2 results show that the FCNx achieves better free navigation space segmentation accuracy than the baseline benchmarks networks with computational complexity of 185 ms. We also compare our proposed network with two baseline benchmarks and show their detection accuracy/cross entropy loss over ten training cycles as shown in Figure 13.

**Figure 14:**
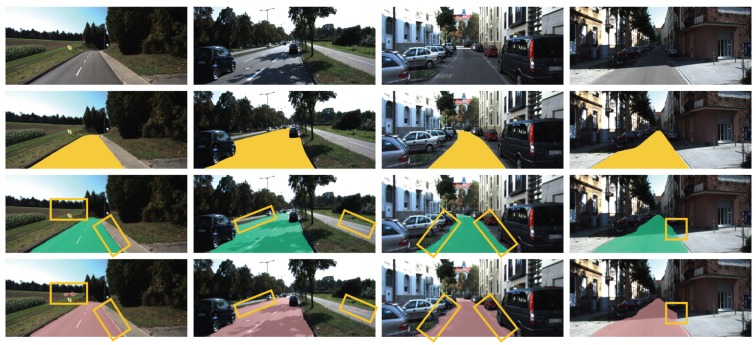
Results of semantic segmentation of the road. First row: some examples of road scenes in various situations. From left to right: Highway clear weather no traffic, highway shadow mix with traffic, urban dim with cars parked, and urban shadow mix with cars parked. Second row: the corresponding road ground truth annotations in yellow overlaid over the original image. Third row: road segmentation by our model FCNx. Row four: road segmentation by UC Berkeley FCN8.

**Figure 15:**
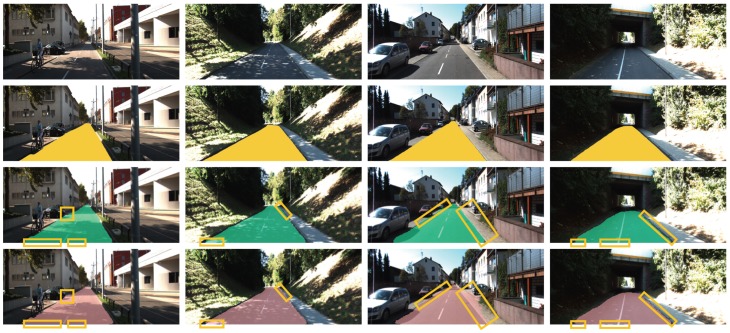
Results of semantic segmentation of the road. First row: some examples of road scenes in various situations. From left to right: City shadow mix scene with moving bicyclist and vehicle, back-road shadow mix with no traffic, urban city road clear sky with cars parked, tunnel dim with no traffic. Second row: the corresponding road ground truth annotations in yellow overlaid over the original image. Third row: road segmentation by our model FCNx. Row four: road segmentation by UC Berkeley FCN8.

Next, we show the results of our FCNx network. We tested our FCNx network on approximately 3000 road scenes and show eight of the more challenging scenes along with the ground truth and the results in Figure 14 and Figure 15. In all scenes shown, by visual examination we can see that our model (row three) performs the road segmentation better and closer to the ground truth (row two) than the benchmark network FCN8 (row four). In Figure 14, we show four scenes from left to right: highway clear weather with no traffic, highway shadow mix with traffic, urban dim with cars parked, and urban shadow mix with cars parked. Our FCNx segmentation was more smooth at segmenting road border lines. For example, in Figure 14, it classified parts of the right-side parking area and right-side sidewalk as the road in the row four columns three and four images, respectively. It also showed some difficulty with False Positives (FP). For example, it falsely classified parts of the left-side grass area and parts of the bicycle path on the right side as the road in the row four column one and two images, respectively.

Also, in Figure 15, we show four scenes from left to right: city shadow mix scene with moving bicyclist and vehicle, back-road shadow mix with no traffic, urban city road clear sky with cars parked, tunnel dim with no traffic. We again see that our network works better at the borders of the road and distinguishing between side walk and road at both near field road and the far field. This can be attributed to sharing, additional filtering before fusion of the encoder feature maps at different decoder levels. In all the eight scenes we highlight the areas our FCNx network performs a better pixel classification and road segmentation with yellow bounding boxes.

**Figure 16:**
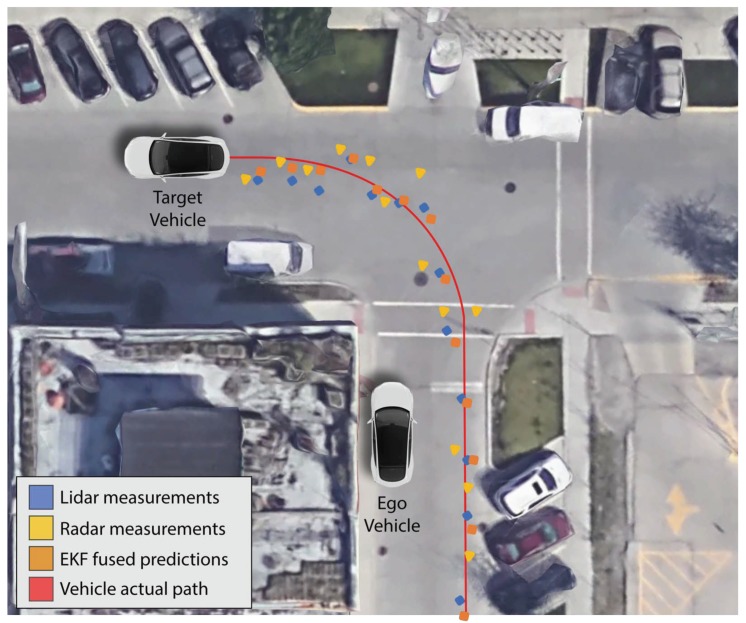
Results of EKF estimated state predictions of a target vehicle from sensors on an ego vehicle. The blue points indicate LiDAR measurements of the target vehicle. The yellow points indicate radar measurements of the target vehicle. The red path shows the actual path the target vehicle traveled, and the orange points show the EKF fused predictions from both LiDAR and radar measurements.

Results of the Extended Kalman filter estimation for a single object tracking are shown in Figure 16. The purpose is to show an example scenario and the feasibility of implementing object tracking using our LiDAR and radar sensor data via a cost-effective embedded computer. Detailed multi-object tracking, track management, data association for more challenging scenarios are not the scope of this paper. The red line shows the actual path of the vehicle we are tracking and the blue and yellow points are the LiDAR and radar measurements, respectively. The orange points are the EKF fused output. The EKF fused predictions are closer to the actual path than individual LiDAR and radar measurements.

We evaluate the performance of the Extended Kalman filter predictions by measuring the root mean square error. RMSE measures how well our prediction values fit the actual path of the target vehicle. RMSE values range from zero to positive infinity depending the data and lower RMSE shows better accuracy. In our simple left-turn scenario experiment (Figure 16), we achieve RMSE of 0.065 and 0.061 (less than 0.1) for the x and y-position of our tracked target vehicle, which shows an improvement over individual LiDAR and radar sensors.

We show the results of each fusion method in our hybrid sensor fusion pipeline. This information gives our AV a detailed map of the environment, which is then sent to the path planning and controls stack of the automated driving pipeline. Having a clear understanding of the surrounding environment can result in optimal decision making, and generating optimal control inputs to the actuators (accelerator, brakes, steering) of our AV.

Finally, we comment on the robustness of our proposed fusion framework in case of sensor failure by discussing the following sensor-failure scenarios:

1. Camera sensor failure: In this case, the pipelines free navigation space segmentation is affected, but it is still able to properly segment using LiDAR depth map alone. Detecting and tracking objects is unaffected and done via the late fused (object level) LiDAR and radar data with the EKF algorithm.
2. Radar sensor failure: In this case, LiDAR can measure the speed of the objects via the range and ToF measurements. However this is slightly less accurate, but still usable for EKF to track the state of objects detected. The road segmentation and object classification remain unaffected.
3. LiDAR sensor failure will not fail the framework. Since the camera and radar essentially are doing the segmentation and tracking tasks on their own, not having LiDAR data will not completely derail or disturb the fusion flow. However, not having LiDAR depth map or point cloud position coordinates information can reduce the segmentation quality as well as tracking accuracy.

In all three scenarios, corrupted inputs can potentially influence the quality of both segmentation, detection and tracking. A concrete analysis of robustness is left for future work.

## 7. Conclusions

The results of each fusion method in our hybrid sensor fusion algorithm give our autonomous vehicle (AV) a detailed map of the environment. Having a clear understanding of the surrounding environment can result in optimal decision making, and generation of optimal control inputs to the actuators (accelerator, brakes, steering) of our AV. In this paper, a new sensor fusion framework configuration is proposed and we demonstrate successful real-time environment perception in a vehicle. Appropriate combinations of Vision + LiDAR and LiDAR + Radar sensor fusion is successfully demonstrated, using a hybrid pipeline: the proposed FCNx and the classic EKF. The algorithm uses our proposed FCNx deep learning framework and is implemented in an edge-computing device in real time using actual sensor data. The proposed solution and implementation enables fusion of camera, radar and LiDAR for a cost-effective embedded solution. For future work, cloud computing and edge computing coordination would be the next step to further enhance this framework. 

## Figures and Tables

**Table 1:** Comparison of our FCNx architecture with FCN8 and U-Net architectures.

Network	Architecture	Skip Connection Operation	Residual Connections
FCN8 [23]	Asymmetrical encoder-decoder base	Skip connections between the encoder and the decoder using element-wise sum operator	Three convolution groups for three downsampling layers before the upsampling path	
U-Net [43]	Symmetrical encoder-decoder base	Skip connections between the encoder and the decoder using concatenation operator	All downsampling layers information is passed to the upsampling path
FCNx (Our model)	Asymmetrical encoder-decoder base	Skip connections between the encoder and the decoder using element-wise sum operator	Four convolution groups for three downsampling layers before the upsampling path

**Table 2:** Comparison of our architecture performance with FCN8 and U-Net networks.

Method	Cross Entropy Loss %	Inference Time (ms)
FCN8 [23]	6.8	∼180
U-Net [43]	7.2	∼92
FCNx (Our model)	3.6	∼185
